# Generation of iPSC lines (KAIMRCi003A, KAIMRCi003B) from a Saudi patient with Dravet syndrome carrying homozygous mutation in the CPLX1 gene and heterozygous mutation in SCN9A

**DOI:** 10.1007/s13577-023-01016-z

**Published:** 2023-12-19

**Authors:** Maryam Alowaysi, Mohammad Al-Shehri, Amani Badkok, Hanouf Attas, Doaa Aboalola, Moayad Baadhaim, Hajar Alzahrani, Mustafa Daghestani, Asima Zia, Khalid Al-Ghamdi, Asayil Al-Ghamdi, Samer Zakri, Sihem Aouabdi, Jesper Tegner, Khaled Alsayegh

**Affiliations:** 1 King Abdullah International Medical Research Center (KAIMRC), King Abdulaziz Medical City, King Saud Bin Abdulaziz University for Health Sciences, Jeddah, Saudi Arabia; 2 Department of Pathology and Laboratory Medicine, Ministry of the National Guard-Health Affairs, Jeddah, Saudi Arabia; 3 Biological and Environmental Science and Engineering Division, King Abdullah University of Science and Technology (KAUST), Thuwal, Saudi Arabia; 4 Forensic Laboratories, Criminal Evidence Department, Jeddah, Saudi Arabia; 5 Computer, Electrical and Mathematical Sciences and Engineering Division, King Abdullah University of Science and Technology (KAUST), Thuwal, Saudi Arabia

**Keywords:** iPSC, CPLX1 variant, SCN9A variant, Dravet syndrome, Epileptic encephalopathy

## Abstract

The most prevalent form of epileptic encephalopathy is Dravet syndrome (DRVT), which is triggered by the pathogenic variant SCN1A in 80% of cases. iPSCs with different SCN1A mutations have been constructed by several groups to model DRVT syndrome. However, no studies involving DRVT-iPSCs with rare genetic variants have been conducted. Here, we established two DRVT-iPSC lines harboring a homozygous mutation in the CPLX1 gene and heterozygous mutation in SCN9A gene. Therefore, the derivation of these iPSC lines provides a unique cellular platform to dissect the molecular mechanisms underlying the cellular dysfunctions consequent to CPLX1 and SCN9A mutations.

**Supplementary Information:**

The online version contains supplementary material available at 10.1007/s13577-023-01016-z.

## Introduction

Dravet syndrome (DRVT, OMIM 607208) is a severe form of developmental and epileptic encephalopathy associated with poor prognosis over the long term and pharmaco-resistance (DRVT, OMIM 607208) [1, 2]. It is frequently triggered by fever during the first year of life in healthy infants [3]. DRVT clinical manifestations include seizures, developmental delay, ataxia, and cognitive impairment, suggesting pathophysiological processes that impair brain development [4–6]. Mutations in the SCN1A gene (Sodium Voltage-Gated Channel Alpha Subunit 1; OMIM 182389) account for 80% of cases [7–12]. SCN1A encodes the voltage-gated sodium channel Nav1.1, which regulates the production and transmission of neuronal action potentials in the central nervous system (CNS) [13, 14]. Additionally, rare genomic variants, such as SCN9A, PCDH19, SCN2A, SCN8A, SCN1B, GABRA1, GABRB3, GABRG2, KCNA2, CHD2, CPLX1, HCN1A, and STXBP1, have been associated with DRVT-like phenotypes [15–18]. A family with autosomal dominant febrile seizures (FS) or genetic epilepsy with febrile seizures plus (GEFS+) has been identified for carrying a sodium channel SCN9A mutation [17]. Moreover, the small presynaptic protein, Complexin 1, is encoded by the CPLX1 gene and forms a soluble N-ethylmaleimide-sensitive factor-attachment protein receptor (SNARE) complex in CNS [19]. CPLX1 regulates the synaptic vesicles exocytosis, and its pathogenic mutations cause several neurological disorders and malignant epilepsy [20, 21].

Although the molecular pathway underpinning SCN1A mutations may be well studied, our knowledge of the pathophysiology of other genes in DRVT remains elusive. One of the main hurdles in understanding the mechanism of epileptic encephalopathies is the lack of an in vitro model that accurately recapitulate the complexity of these disorders, including DRVT [22]. Epilepsy patient-specific-induced pluripotent stem cells (iPSCs) represent a distinctive biological platform to study disease onset and progression [22, 23]. Several groups have modeled DRVT in vitro by reprogramming patients’ fibroblasts into iPSCs carrying distinct mutations in SCN1A [24–26]. Transcriptomic profiling from differentiated DRVT-iPSCs into neural progenitor cells and GABAergic cells represented gene expression signatures of “Prefrontal Cortex,” “Fetal Brain,” “Dentate Gyrus,” and “Superficial Dorsofrontal Area,” brain regions that are correlated with clinical features in DRVT [25]. Furthermore, electrophysiological aberrations in DRVT-iPSC GABA lines are consistent with reduced sodium current density and decreased excitability of inhibitory interneurons, as previously reported in a DRVT animal model [25, 27]. These combined results indicate that DRVT-iPSC lines are appropriate model for studying the neuro-pathophysiological processes underlying DRVT.

In this study, we generated iPSCs from DRVT patient with homozygous mutation in the CPLX1 gene and heterozygous mutation in SCN9A using non-integrating episomal expression of the reprogramming factors OTC3/4, L-MYC, LIN28, SOX2, KLF4, and mp53DD. Our established DRVT-iPSC lines possess bona fide characteristics of embryonic stem cells and constitute a primary cellular model to interrogate the molecular basis of rare genetic variants in DRVT.

## Materials and methods

### Patient recruitment and ethical approval

This study was approved by the institutional review board (IRB) and research ethics committee of KAIMRC (NRJ22J/060/03). The patient is a 7-year-old female diagnosed with DRVT syndrome carrying C.4G > A (P.Glu2Lys) homozygous mutation in CPLX1 and C.3332-3346del (P.Ser1111_Glu1115del) heterozygous mutation in SCN9A identified by whole exome sequencing (WES). The patient's parents gave their consent via an informed consent form (ICF), which was used to collect and process the patient's sample.

### PBMCs isolation and enrichment of erythroid progenitors

According to the manufacturer's instructions, peripheral blood from Saudi patients was drawn into a blood collection tube containing EDTA and processed with the RosetteSep™ Human Progenitor Cell Basic Pre-Enrichment antibody cocktail (Stem Cell Technologies Catalog #15226). 1 million cells were cultivated for 8 days after PBMC separation and isolation in StemSpan™ SFEM II medium (Stem Cell Technologies Catalog #09605) with 1X StemSpan™ Erythroid Expansion Supplement (Stem Cell Technologies Catalog #02692).

### Transfection of erythroid progenitor cells

Episomal iPSC Reprogramming Kit (Thermofisher Catalog #A15960) was used to reprogram expanded erythroid progenitor cells. Three pulses at 1650 V and a pulse width of 10 ms were used with 1 μg of each episomal vector (Neon Transfection System, Thermofisher). The emerging ESC-like colonies were then manually selected for transfer into 48-well plates coated with Geltrex™ LDEV-Free Reduced Growth Factor Basement Membrane Matrix (Thermofisher Catalog #A1413201) with mTeSR™ Plus media at 37 °C with 5% CO_2_ and 20% O_2_. iPSCs were dissociated using Gentle Cell Dissociation Reagent (Stem Cell Technologies Catalog #100-0485) and passaged using 1:20–1:30 splitting ratio.

### Immunocytochemistry

Cells were first fixed for 15 min in 4% (w/v) paraformaldehyde, then permeabilized for 10 min in PBS containing 0.1% (v/v) Triton X-100 and blocked for 45 min in PBS containing 1% Gelatin. Primary antibodies were incubated overnight at 4 °C before they were probed with the proper secondary antibodies for one hour at room temperature (Table 1). Primary and secondary antibodies were reconstituted in PBS containing 0.2% gelatin. DAPI nuclear staining at 1 μg/mL was used to stain the nuclei. The staining was visualized under Zeiss LSM 880 Airyscan confocal laser scanning microscope using a 20× oil objective (Zeiss).

**Table 1:** List of antibodies and primers used in this study

Antibodies and stains used for immunocytochemistry/flow-cytometry			
	Antibody	Dilution	Company Cat # and RRID
Pluripotency Markers	Rabbit anti-OCT4	1:100	Abcam Cat# ab200834 RRID# AB_2924374
	Goat anti-NANOG	1:100	Abcam Cat# ab109250 RRID# AB_10863442
	Goat anti-SOX2	1:100	Abcam Cat# ab93689 RRID# AB_10562630
Secondary antibody	Goat anti-Rabbit Secondary Antibody, Alexa Fluor 488	IF 1:200 Flow Cyt 1:2000	Abcam Cat#: ab150077 RRID# AB_2630356

Primers and oligonucleotides used in this study		
	Target	Forward/reverse primer (5′–3′)
Differentiation markers RT-qPCR	BRACHYURY or TBXT	TAAGGTGGATCTTCAGGTAGC CATCTCATTGGTGAGCTCCCT
	CDX2	GACGTGAGCATGTACCCTAGC GCGTAGCCATTCCAGTCCT
	NESTIN	CTGCTACCCTTGAGACACCTG GGGCTCTGATCTCTGCATCTAC
	PAX6	TGGGCAGGTATTACGAGACTG ACTCCCGCTTATACTGGGCTA
	SOX17	GCATTCTGGAATGAGCCTACT GGGCAGGTCAAGCTTATGAT
	GATA4	CGACACCCCAATCTCGATATG GTTGCACAGATAGTGACCCGT
House-Keeping Genes (qPCR)	GAPDH	GGAGCGAGATCCCTCCAAAAT GGCTGTTGTCATACTTCTCATGG
Mutation analysis	CPLX1 SCN9A	CTCACGAAGGACTTCCCAG GTAAAGCCTACGCCAGAGTG TAGGGTATAGGTTGTCCTC CATGGAATGGAGTCTTCTG
EBNA-1	pEP4-SF2-oriP pEP4-SR2-oriP	ATC GTC AAA GCT GCA CAC AG CCC AGG AGT CCC AGT AGT CA

### Quantitative reverse transcription PCR (RT-qPCR)

RNeasy Kit (Qiagen Catalog #74104) was used to extract total RNA, and SuperScript™ III First-Strand Synthesis (Thermo Fisher Scientific Catalog #18080051) was used to reverse-transcribe the extracted total RNA. As previously explained [28], the RT-qPCR test was performed using FastStart SYBR Green Master Mix (ROCHE).

### In vitro differentiation

The STEMdiff™ Trilineage Differentiation Kit (Stem Cell Technologies Catalog #05230) was used to differentiate the generated DRVT-iPSCs into three germ layers.

### Flow cytometry analysis

The BD IntraSure™ Kit was used for permeabilizing and staining for Intracellular Markers (BD Biosciences Catalog #641778). Briefly, 4 × 10^5^ cells were fixed using Reagent A for 10 min. Primary antibodies (Table 1) were diluted in Reagent B and incubated on ice for 30 min. Secondary antibodies were diluted with PBS and incubated for 30 min at room temperature. FACS samples were analyzed on BD FACS ARIA cell sorter. FITC-positive cells were measured in stained vs unstained cells.

### Karyotype analyses

For G-banding karyotyping, iPSC lines were treated with 0.3 g/mL KaryoMAX™ Colcemid™ for 15 min, dissociated by TrypLE, and incubated in hypotonic solution (75 mM potassium chloride) at 37 °C for 20 min. After that, iPSCs were preserved at 4 °C after being fixed in a methanol/glacial acetic acid 3:1 mixture. Pathology and laboratory medicine (Ministry of the National Guard—Health Affairs) performed karyotyping on at least 50 metaphases.

### Plasmids screening

Utilizing the All Prep DNA/RNA/Mini Kit (Qiagen Catalog #80204), DNA was extracted according to manufacturer instructions. PCR was carried out using EBNA-1 primers that identify all five episomal plasmids (expected size 666 bp) (Thermo Fisher Scientific Catalog #A15960).

### Short tandem repeat (STR)

Following the manufacturer's instructions, genomic DNA was extracted from PBMCs and DRVT-iPSC lines DNeasy Blood&Tissue kit and AllPrep DNA/RNA/Mini Kit, respectively. The PowerPlex^®^ 16 System (Promega) kit was used to amplify 15 STR loci and Amelogenin. On a 3130 Genetic Analyzer from Applied Biosystems, PCR amplicons were resolved. GeneMapper ID-X Software, version 1.4 was used to gather and evaluate the data.

### Mycoplasma detection

Mycoplasma contamination was assessed using LookOut^®^Mycoplasma qPCR Detection (SIGMA).

### Statistical analysis

RT-qPCR data are represented as mean ± standard deviation (SD). Statistical significance was determined in Student’s *t*-test (unpaired; two-tailed). A Bonferroni correction was applied to the *p* value from multiple comparisons. *p < 0.05.

## Results

### Clinical information and mutation analysis

**Fig. 1:**
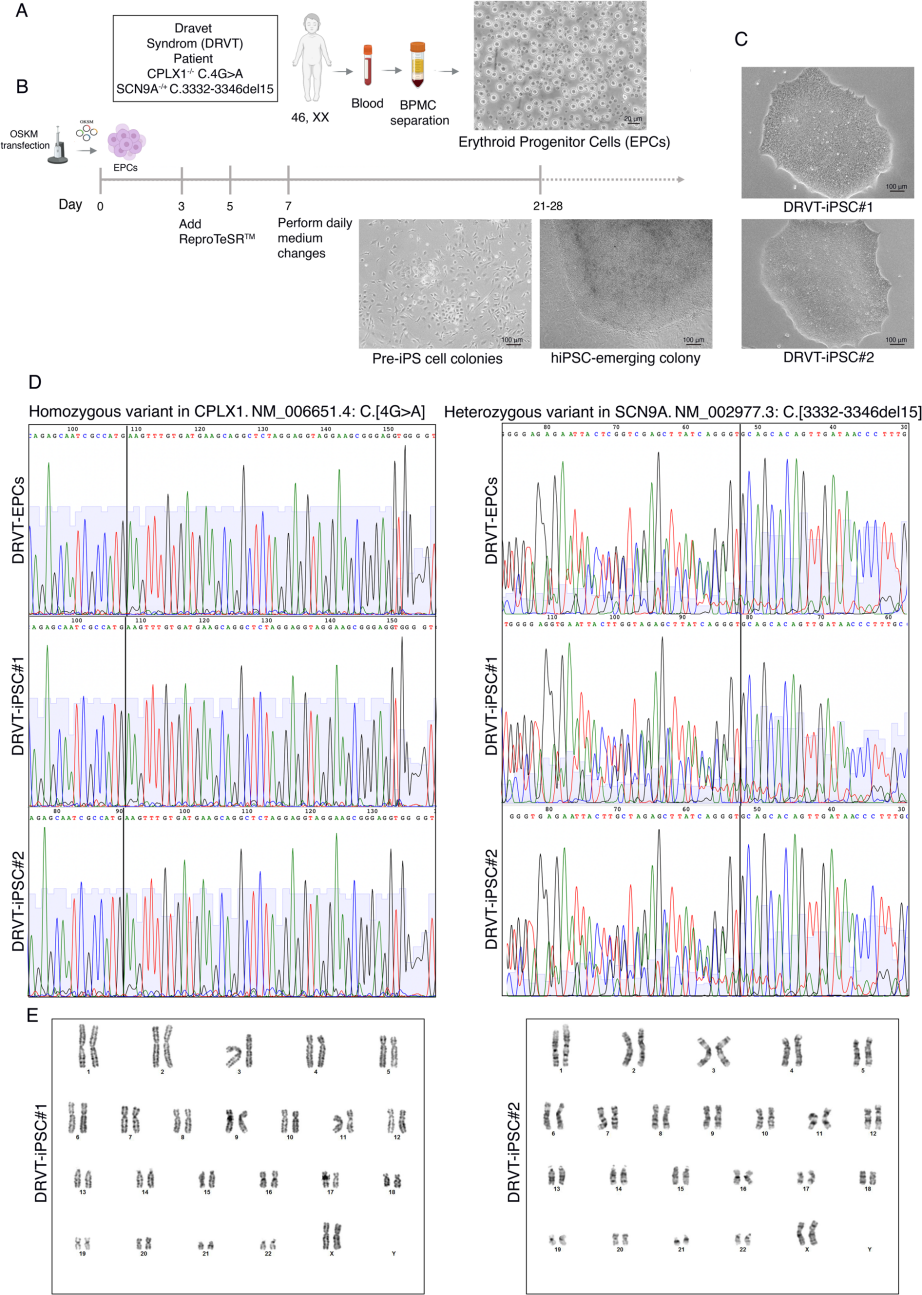
Cellular reprogramming and Generation of DRVT-iPSCs. **A** Collecting a sample of 10 ml peripheral blood from DRVT syndrome patient and expanding erythroid progenitor cells (EPCs) for eight days. **B** ReproTeSR™ and the episomal reprogramming process are represented schematically. During reprogramming (days 11–28), phase-contrast pictures of the mesenchymal–epithelial transition and the emergence of colonies were captured. **C**. Representative images of DRVT-iPSC clones show defined borders and compact morphology. **D** Sanger sequencing confirms the mutations in SCN9A and CPLX1 in the patient's peripheral blood cells as well as in the DRVT-iPSC lines. **E** Karyotypes for DRVT-iPSCs exhibit normal chromosomal content 46, XX by representative G-banded karyotype analyses

The 7-year-old female patient was diagnosed with global developmental delay, seizers since in infancy, and choreoathetotic movements. Molecular genetic analysis for whole exome sequencing (WES) showed positive family history suggestive of an autosomal recessive pattern of inheritance. In addition, WES of the patient blood sample identified heterozygous mutation c.3332_3346del p.(Ser1111_Glu1115del) in SCN9A gene. This mutation leads to an in-frame deletion of 15 bps in exon 18 (NM_002977.3), which causes the loss of 5 amino acid residues. Further analysis unraveled homozygous variant c.4G > A p.(Glu2Lys) in the CPLX1 gene. This variant leads to an amino acid exchange in exon 1 (NM_006651.4). Both mutations were confirmed in the patient's peripheral blood cells as well as in the DRVT-iPSC lines by Sanger sequencing (Fig. 1D).

### The generation of integration-free DRVT-iPSC lines

Initial phone contact with the donor’s parent resulted in the scheduling of an in-person interview after receiving approval. Following the signature on the informed consent form, a sample of 10 mL peripheral blood was drawn and erythroid progenitor cells (EPCs) were cultured for eight days (Fig. 1A). Due to their lack of genetic mutations and genomic structural variation, including the absence of TCR/BCR gene recombination found in T cells, EPCs were selected for reprogramming [40]. We, therefore, established two DRVT-iPSC lines using a non-integrative and virus-free reprogramming approach as previously described [29]. Briefly, the episomal vectors encoding OCT4, SOX2, KLF4, L-MYC, LIN28A, dominant-negative form of TP53, and EBNA1 were delivered to EPCs by electroporation (Fig. 1B). We identified emerging embryonic stem cell (ESC)-like colonies with typical ESC morphological characteristics (i.e., distinct borders, bright centers, tight-packed cells, and a high nucleus-to-cytoplasm ratio) after around 20 days (Fig. 1C). The derived iPSC lines were picked, expanded in feeder-free condition, and cryopreserved in KAIMRC facility. We thawed two DRVT-iPS clones for downstream testing.

We evaluated the genomic integrity and confirmed the genetic compatibility of the generated DRVT-iPSCs and EPCs. A normal female chromosomal number and structure has been shown by G-banding analysis (Fig. 1E). The matched identities of the isolated iPS lines and the donor EPCs were validated by a short tandem repeats (STR) assay (Fig. S1B). Moreover, mycoplasma testing showed that the generated iPSC lines are mycoplasma-free (Fig. S1A).

### Characterization of self-renewal and pluripotency

**Fig. 2:**
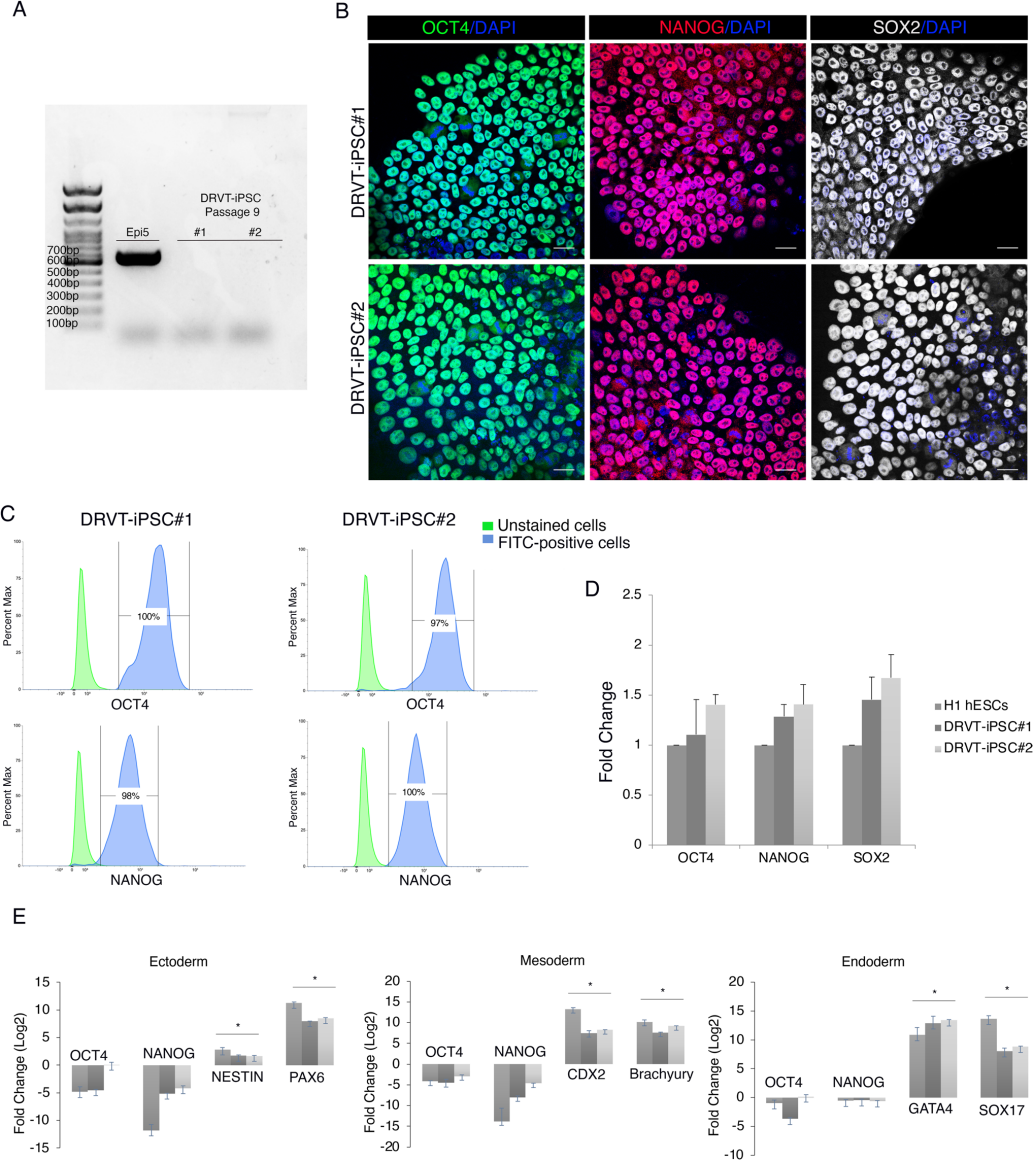
Pluripotency characterization of the derived DRVT-iPSCs. **A** The five episomal plasmids were shown to be absent in the generated DRVT-iPSCs by endpoint PCR. **B** immunofluorescence staining of the pluripotency markers OCT4 (green), NANOG (red), and SOX2 (yellow), Nuclei were stained with DAPI (blue). Scale bar = 20 μm. **C** Flow cytometry histograms of OCT4, NANOG, and SOX2 in DRVT-iPSCs. **D** Graph showing mRNA expression levels of pluripotency markers for the indicated iPSC lines presented as fold change relative to H1 hESC. Bars are median ± std of 3 biological replicates for each sample. **E** Graphs showing mRNA expression levels of the lineage-specific markers for the three germ layers Mesoderm (CDX2 and Brachyury), Endoderm (GATA4 and SOX17), and Ectoderm (NESTIN and PAX6) presented as fold change relative to undifferentiated cells. Bars are median ± std of 3 biological replicates for each sample. Student’s *t* tests, *p < 0.05

The slow removal of cellular episomal vectors from DRVT-iPSC lines was achieved after culturing the cells for nine passages (Fig. 2A). As a result, we rigorously evaluated the pluripotency using multiple approaches. We assessed the endogenous expression of pluripotency markers of OCT4, NANOG, and SOX2, by immunofluorescence and RT-qPCR (Fig. 2B; Fig. 2D). Flow cytometry histograms demonstrated that > 97% of cells stained positively for OCT4 and > 98% for NANOG (Fig. 2C). Direct in vitro differentiation to the three germ layers, mesoderm, endoderm, and ectoderm was used to demonstrate the tri-lineage differentiation capacity. We observed a down-regulation of OCT4 and NANOG and an upregulation of germ layer-specific markers (Fig. 2E). The positive expression of the neural progenitor markers of the central nervous system NESTIN and PAX6 indicated ectodermal differentiation. We demonstrated an upregulation of Brachyury, a member of the T-box family, and CDX2, a caudal-type homeobox protein 2, which indicated an early determination of mesoderm. We further examined the presence of the endodermal marker SRY-Box Transcription Factor 17 SOX17 and zinc-finger transcription factor GATA4 in DRVT-iPSC lines and H1 hESC positive control (Fig. 2E). However, the fold change in NANOG is not substantial during endoderm lineage. Teo et al. 2011 demonstrated that while OCT4 and SOX2 prevent DE differentiation of hESCs, NANOG is required to initiate EOMESODERMIN (EOMES) expression, which then interacts with SMAD2/3 to activate the transcriptional network that directs endoderm formation [45].

Subsequently, the derived DRVT-iPSC lines were verified for pluripotency and registered in the Human Pluripotent Stem Cell Registry https://hpscreg.eu/user/cellline/edit/KAIMRCi003-A; https://hpscreg.eu/user/cellline/edit/KAIMRCi003-B.

## Discussion

In the last decade, the seminal discovery of cellular reprogramming and the generation of iPSCs have been widely utilized to model diseases “in a dish” and hold promise for applied biology and regenerative medicine [23, 30]. The characteristics of iPSCs resemble those of embryonic stem cells, including their morphology, self-renewal, gene expression, and the capacity to differentiate into virtually any cell type of the body [23]. These changes are accompanied by transient expression of the pluripotency transcription factors NANOG, OCT3/4, SOX2, KLF4, c-MYC, and LIN28 [31–37]. These factors exert a dual role by promoting the expression of pluripotency-associated genes in a self-regulatory loop and silence somatic genes [38, 39]. Although iPSCs can be derived from multiple sources of somatic cells, EPCs are chosen for their lack of chromosomal aberrations and genomic DNA mutations [40]. We found that eight days of expansion in erythroid expansion medium, yielded 69% of cells positive for CD71 + CD235a + erythroid cell surface markers [41]. The non-viral, non-integrating episomal plasmid-based reprogramming technique is practically applicable for generating clinical grade-iPSCs [42]. Vectors containing oriP and EBNA-1, based on the Epstein–Barr Nuclear Antigen-1, have shown the ability to create iPSCs highly effectively with a single transfection [43].

Even though multiple groups have generated iPSCs with various SCN1A mutations to model DRVT syndrome, no studies have been conducted employing uncommon genetic variants [24–26]. Pathogenic variants in the SCN9A gene have been associated with several autosomal dominant conditions, including familial febrile seizures 3B (613863) and generalized epilepsy with febrile seizures plus type 7 [16]. Moreover, mutation in CPLX1 gene is causative for autosomal recessive developmental and epileptic encephalopathy 63. DEE63 is a neurologic disorder characterized by early-onset refractory infantile spasms and myoclonic seizures in the first months to years of life [21, 44].

Intriguingly, the differentiation of DRVT-iPSC into neuronal subtypes has yielded important mechanistic understandings of the disorder. For example, studies have shown that DRVT-iPSC-derived medial ganglionic eminence (MGE)-like inhibitory neuron reduced the action potential frequency compared to those in controls [24]. In addition, the transcriptome analysis of DRVT-iPSC-derived NPCs and GABA cells compared to controls, identified unique dysregulations of genes for chromatin structure, mitotic progression, neuronal plasticity, and excitability [25]. Therefore, future research involving the differentiation of DRVT-iPSC#1 and iPSC#2 into neural cells, gene expression profiling, and functional prosperity, will provide valuable insights into the disorder. In parallel, we will pursue an isogenic design of SCN9A and CPLX1 knockouts in H9 hESC using CRISPR/Cas9 to validate results obtained from DRVT-derived neural cells. In this isogenic setting, DRVT transcriptional alterations will be corroborated with dysregulated genes putatively attributable to SCN9A and CPLX1 deficiencies in disease-relevant tissues. Hence, their usefulness extends from in vitro disease modeling to drug screening, paving the way for unraveling disease mechanisms and accelerating the discovery of novel therapeutic targets for the treatment of Dravet Syndrome.

### Supplementary Information

Below is the link to the electronic supplementary material.

Supplementary file1 Fig. 1S: A. RT-qPCR showing negative mycoplasma test in DRVT-iPSC lines. B. Short Tandem Repeat (STR) profiling guaranteed the genetic identity between the established iPSC lines and the donor EPCs. (TIF 164478 KB)

## Data Availability

The data that support the findings of this study are openly available. All characterization data related to this study can be accessed upon reasonable request. Requests for access to this data should be directed to Dr. Khaled Alsayegh, alsayeghk@kaimrc.edu.sa.
